# The plant organellar primase-helicase directs template recognition and primosome assembly via its zinc finger domain

**DOI:** 10.1186/s12870-023-04477-4

**Published:** 2023-10-06

**Authors:** Antolin Peralta-Castro, Francisco Cordoba-Andrade, Corina Díaz-Quezada, Rogerio Sotelo-Mundo, Robert Winkler, Luis G. Brieba

**Affiliations:** 1 Langebio-Cinvestav Sede Irapuato, Km. 9.6 Libramiento Norte Carretera. Irapuato-León, 36821 Irapuato Guanajuato, Mexico; 2 Laboratorio de Estructura Biomolecular, Centro de Investigación en Alimentación y Desarrollo, A.C. Carretera Gustavo Enrique Astiazarán Rosas Núm. 46, Ejido a La Victoria, 83304 Hermosillo, Sonora, Mexico

**Keywords:** RNA polymerase, Primase, Organelle, Flowering plants

## Abstract

**Background:**

The mechanisms and regulation for DNA replication in plant organelles are largely unknown, as few proteins involved in replisome assembly have been biochemically studied. A primase-helicase dubbed Twinkle (T7 gp4-like protein with intramitochondrial nucleoid localization) unwinds double-stranded DNA in metazoan mitochondria and plant organelles. Twinkle in plants is a bifunctional enzyme with an active primase module. This contrast with animal Twinkle in which the primase module is inactive. The organellar primase-helicase of *Arabidopsis thaliana* (AtTwinkle) harbors a primase module (AtPrimase) that consists of an RNA polymerase domain (RPD) and a Zn + + finger domain (ZFD).

**Results:**

Herein, we investigate the mechanisms by which AtTwinkle recognizes its templating sequence and how primer synthesis and coupling to the organellar DNA polymerases occurs. Biochemical data show that the ZFD of the AtPrimase module is responsible for template recognition, and this recognition is achieved by residues N163, R166, and K168. The role of the ZFD in template recognition was also corroborated by swapping the RPDs of bacteriophage T7 primase and AtPrimase with their respective ZFDs. A chimeric primase harboring the ZFD of T7 primase and the RPD of AtPrimase synthesizes ribonucleotides from the T7 primase recognition sequence and conversely, a chimeric primase harboring the ZFD of AtPrimase and the RPD of T7 primase synthesizes ribonucleotides from the AtPrimase recognition sequence. A chimera harboring the RPDs of bacteriophage T7 and the ZBD of AtTwinkle efficiently synthesizes primers for the plant organellar DNA polymerase.

**Conclusions:**

We conclude that the ZFD is responsible for recognizing a single-stranded sequence and for primer hand-off into the organellar DNA polymerases active site. The primase activity of plant Twinkle is consistent with phylogeny-based reconstructions that concluded that Twinkle´s last eukaryotic common ancestor (LECA) was an enzyme with primase and helicase activities. In plants, the primase domain is active, whereas the primase activity was lost in metazoans. Our data supports the notion that AtTwinkle synthesizes primers at the lagging-strand of the organellar replication fork.

**Supplementary Information:**

The online version contains supplementary material available at 10.1186/s12870-023-04477-4.

## Background

A wealth of proteins involved in DNA transactions assemble as modular proteins with domains specialized in DNA binding, catalysis, or protein-protein interactions [1, 2]. Archetypical examples of modular proteins are the bifunctional primase-helicases. These proteins replicate plasmids, viral, and organellar genomes [3, 4]. Bifunctional primase-helicases are oligomeric proteins that assemble as hexamers or heptamers [5–8]. The primase-helicase from bacteriophage T7 is the representative member of this protein family [4]. In hexameric primase-helicases, the helicase domain unwinds double-stranded DNA using nucleotide hydrolysis while the primase domain recognizes a templated sequence to drive RNA synthesis. Primer synthesis by primase-helicases is achieved by the combination of their Zn^++^ finger (ZFD) and RNA polymerase (RPD) domains of the primase module [9, 10]. DNA primases recognize a trinucleotide DNA sequence in which the 3´ base is cryptic, meaning that this base is necessary for template recognition but is not instructional for the identity of the primer [6]. Primases recognize multiple template sequences. For instance, phylogenetically related primase-helicases from bacteriophages T7 and SP6 recognize the 5′-GTC-3′ and 5′-GCA-3′ sequences, respectively, where the underlined nucleotide is cryptic, as mentioned before meaning that this base is necessary for recognition but its complementary base is not copied into the RNA primer [6].

**Fig. 1:**
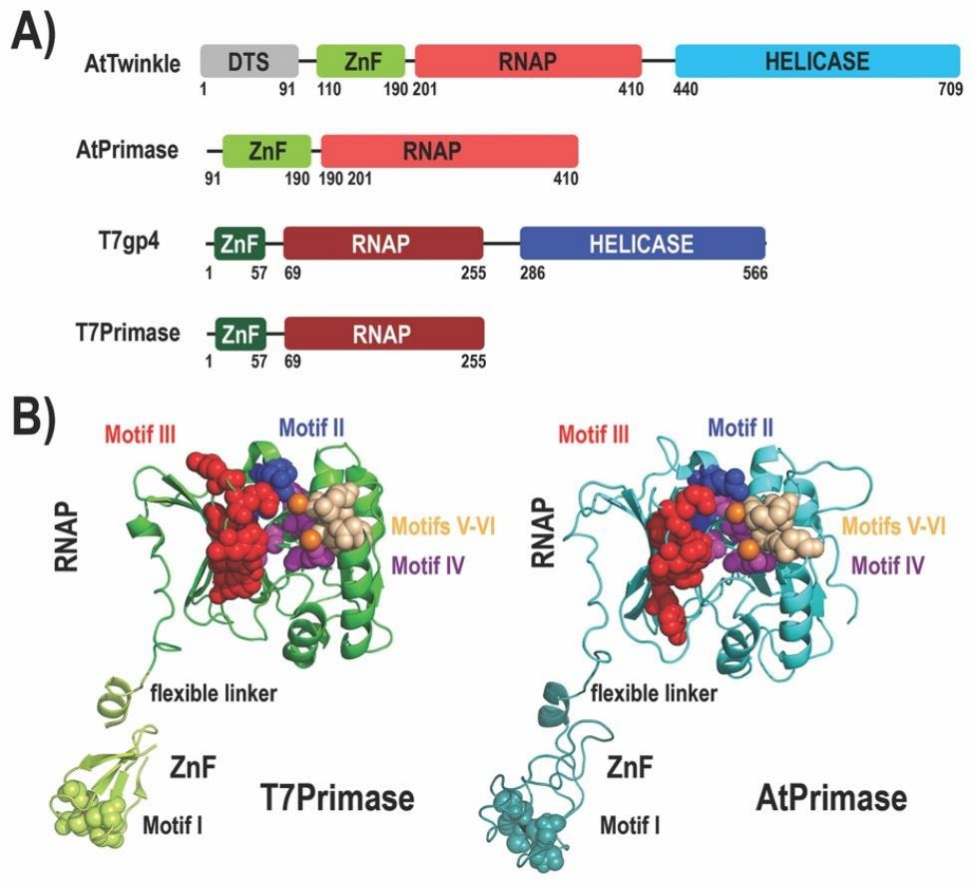
Domain and structural representation of T7 Primase and AtPrimase. **(A)** Domain organization of the hexameric primase-helicases of bacteriophage T7 and *A. thaliana´*s mitochondria. The boundaries of the helicase domains (blue and cyan blocks), primase (green blocks for the ZFD and red blocks for the RNAP domain, respectively) are indicated by numbers. AtTwinkle contains a signal peptide for mitochondrial and chloroplast targeting (grey block) at its N-terminus. **(B)** Crystal structure T7 Primase in comparison to a structural model of AtPrimase. Motifs II to VI that correspond to the RNAP domain are in ball-stick representation. The region corresponding to the Zn^++^ finger is the one that shows the greatest amino acid sequence divergence between primases

The mechanisms for DNA replication in plant mitochondria remains to be elucidated. Several groups debate over a mechanism involving the role of homologous recombination at DNA breaks as starting points for DNA replication and the formation of a T7-like replisome that generates primers for the plant mitochondrial DNAPs [11–14]. Plant mitochondria harbor enzymes capable to execute DNA replication by a T7-like replisome and by homologous recombination [11, 12, 14, 15]. In all eukaryotes, with the exception of metazoans, Twinkle (a T7 gp4-like protein with intramitochondrial nucleoid localization) is predicted to be active as a primase and helicase [16]. During lagging strand DNA synthesis, DNA primases perform multiple functions: (1) recognition of a ssDNA sequence, (2) *de novo* RNA synthesis, and (3) primer transfer to a DNAP. T7 primase-helicase, AtTwinkle, and *Dictyostelium discoideum* (DdTwinkle) are the only characterized enzymes from the primase–helicase family [17–19]. (Fig. 1A). The primase module consists of a ZFD and an RPD connected by a flexible amino acid linker. It is proposed that the ZFD fulfills roles in template recognition, primer stability, and primer transfer [5, 18, 20, 21]. The Twinkle ortholog of the plant model *Arabidopsis thaliana* (AtTwinkle) is active, both as a primase and as a helicase [13, 22, 23]. Contrary to all primases characterized to date, the primase module of AtTwinkle recognizes two nucleotides (instead of one) as cryptic elements. The ssDNA recognition sequence of AtTwinkle is 3´-AGGG/C-5´, in which the 3´-AG-5´pair is cryptic [13]. The ZFD of AtTwinkle displays unique amino acids that possibly interact with the ssDNA template sequence, suggesting that the recognition of two bases as cryptic elements is mediated by its ZFD [13]. Biochemical analysis indicates that template specificity is mediated by the RNAP and ZFD in RNA primases [20]. The “*cis*” model for *de novo* synthesis and ribonucleotide extension postulates that the Zn^++^ finger recognizes the cryptic and the first two template nucleotides, directing the synthesis of the first two ribonucleotides [18]. Thus, in T7 Primase the ZFD is important for template recognition, transfer of the initial diribonucleotide from the RPD, and hand-off of the RNA primer to the T7 DNAP polymerase active site [15, 28–30]. In support of these biochemical studies, the structure of the T7 primosome illustrates that the ZFD of the T7 Primase domain intercalates between the fingers and thumb subdomains of T7 DNAP and that the RPD mainly interacts with the thumb subdomain [24].

To study the possible role of the ZFD in template recognition, we performed a functional analysis of the amino acids that are possibly involved in template recognition and used swapping subdomain experiments between the Zn^++^ fingers of T7 and Arabidopsis primases. Our results are congruent with the existence of a T7-like replisome in plant mitochondria that directs mitochondrial DNA replication and the formation of a T7 primosome to initiate mitochondrial DNA synthesis [12].

## Results

### AtPrimase is a modular protein poised to interact with ssDNA

The RNAP and ZFDs subdomains of T7 primase and AtTwinkle share 26% and 18% amino acid identity, respectively. A structural model of AtPrimase in comparison to T7 primase highlights the conservation of the active site residues in Motifs I to VI **(**Fig. 1B and Fig. 1S).

**Fig. 2:**
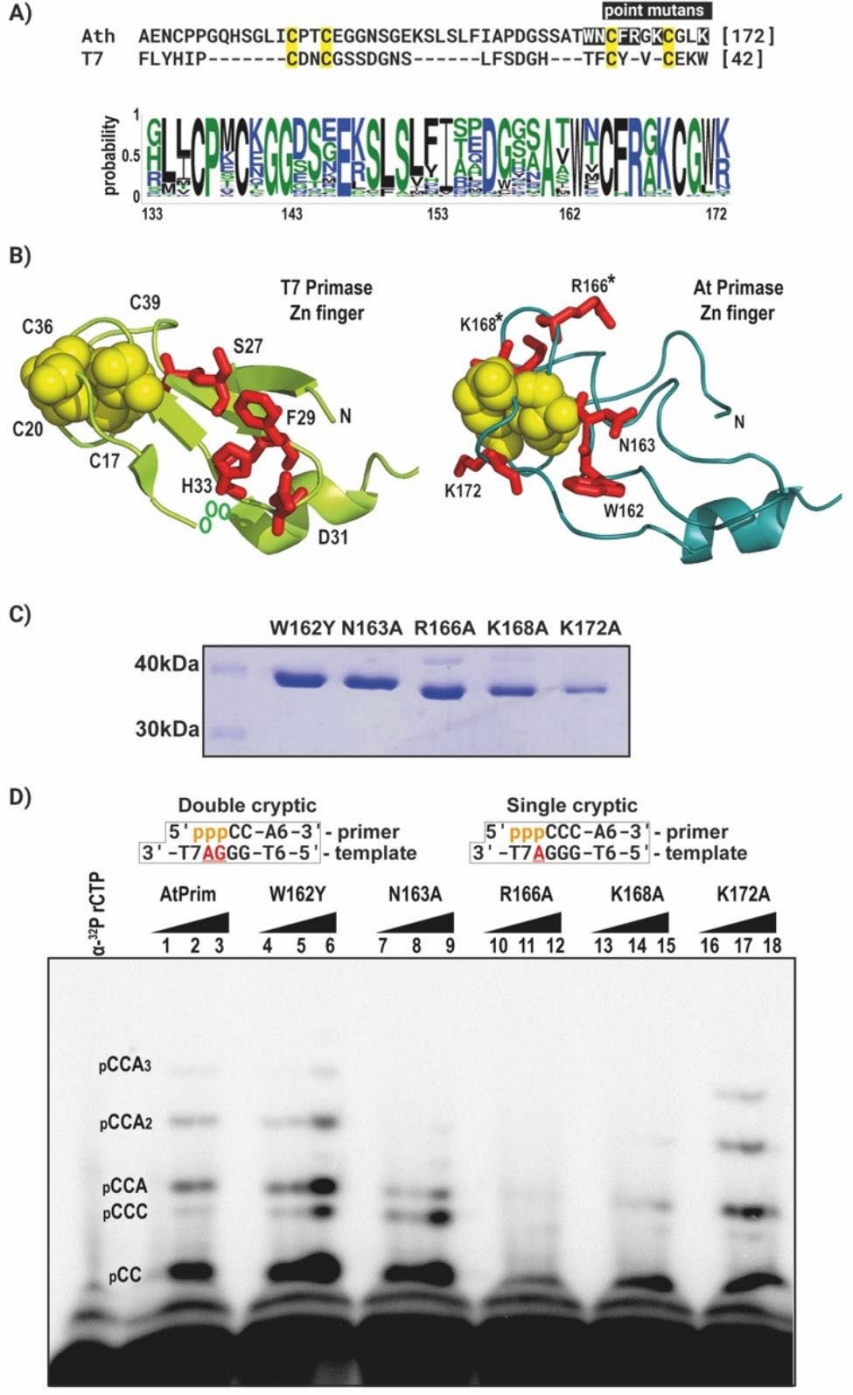
The ZFD is involved in recognition of the cryptic pair within the single-stranded recognition sequence. **A)** Amino acid sequence alignment of the region corresponding to the ZFD of AtPrimase and T7 Primase. The cysteines that coordinate the zinc atom are highlighted in yellow and the residues that were mutated in AtPrimase are highlighted in black. The amino acid sequence logo corresponds to an alignment of the ZFD of plants. **B)** Structural comparison of the analyzed residues in the ZFD model of AtPrimase (red colored) and the structure of the ZFD of T7 primase **C)** SDS-PAGE gel showing purified ZFD mutant proteins. **D)** Denaturing sequencing gel showing ribonucleotide synthesis by wild-type AtPrimase and the analyzed point mutations on a canonical 5’-T_6_GGGAT_7_-3’ substrate, the underlined GA corresponds to the cryptic element of AtPrimase. This substrate drives the synthesis of a 5’-pppCC dimer than can be extended by the incorporation of six adenines to the template thymines. Wild-type AtPrimase (lanes 1 to 3) synthesizes the expected RNA products from a canonical template, while the K166A (lanes 10 to 12) and K168A (lanes 13 to 15) mutants display no activity on this substrate. The K172A mutant (lanes 16 to 18) shows a similar ribonucleotide synthesis with respect to the wild type. Mutant W162Y (lanes 4 to 6) and N163A (lanes 7 to 9) show an increase in the formation of the pCCC triribonucleotide. This product corresponding to the recognition of only one cryptic base

The main difference in primary sequence between the ZFDs of AtTwinkle and T7 primase is the insertion of eight amino acids between the second and third cysteines that coordinate a Zn^++^ atom in the ZFD of AtTwinkle (Fig. 2A). According to several structural function studies, the T7 primase residues D31, S27, F29, H33, K41, and a group of aromatic amino acids (F29, F35, Y37, and W42) interact with the ssDNA template [21, 25–27]. AtPrimase does not retain this group of amino acids **(**Fig. 2A**)**. Additionally, AtPrimase contains a number of residues close to the third and fourth Zn + + finger cysteines that are not conserved with T7 primase, and because of their proximity to amino acids that interact with ssDNA, we hypothesize that these residues have the ability to interact with ssDNA and play a role in ssDNA recognition. Those residues are W162, N163, R166, K168, and K172 **(**Fig. 2B and Fig. 1S).

To investigate this hypothesis, we mutated residue W162 for Try and the other residues for alanine (Fig. 2C) and evaluated their ribonucleotide synthesis on a canonical ssDNA template (Fig. 2D). AtPrimase-W162Y and AtPrimase-K172A synthesize RNA products that exhibited a similar pattern to the products synthetized by wild-type AtPrimase (Fig. 2D). Mutant AtPrimase-N163A, increases the synthesis of the pCCC ribonucleotide and AtPrimase-K168A strongly decreases ribonucleotide incorporation, whereas the AtPrimase-R166A mutant is largely inactive **(**Fig. 2D**).**

### Residues R166 and K168 are directly involved in recognition of the cryptic pair

**Fig. 3:**
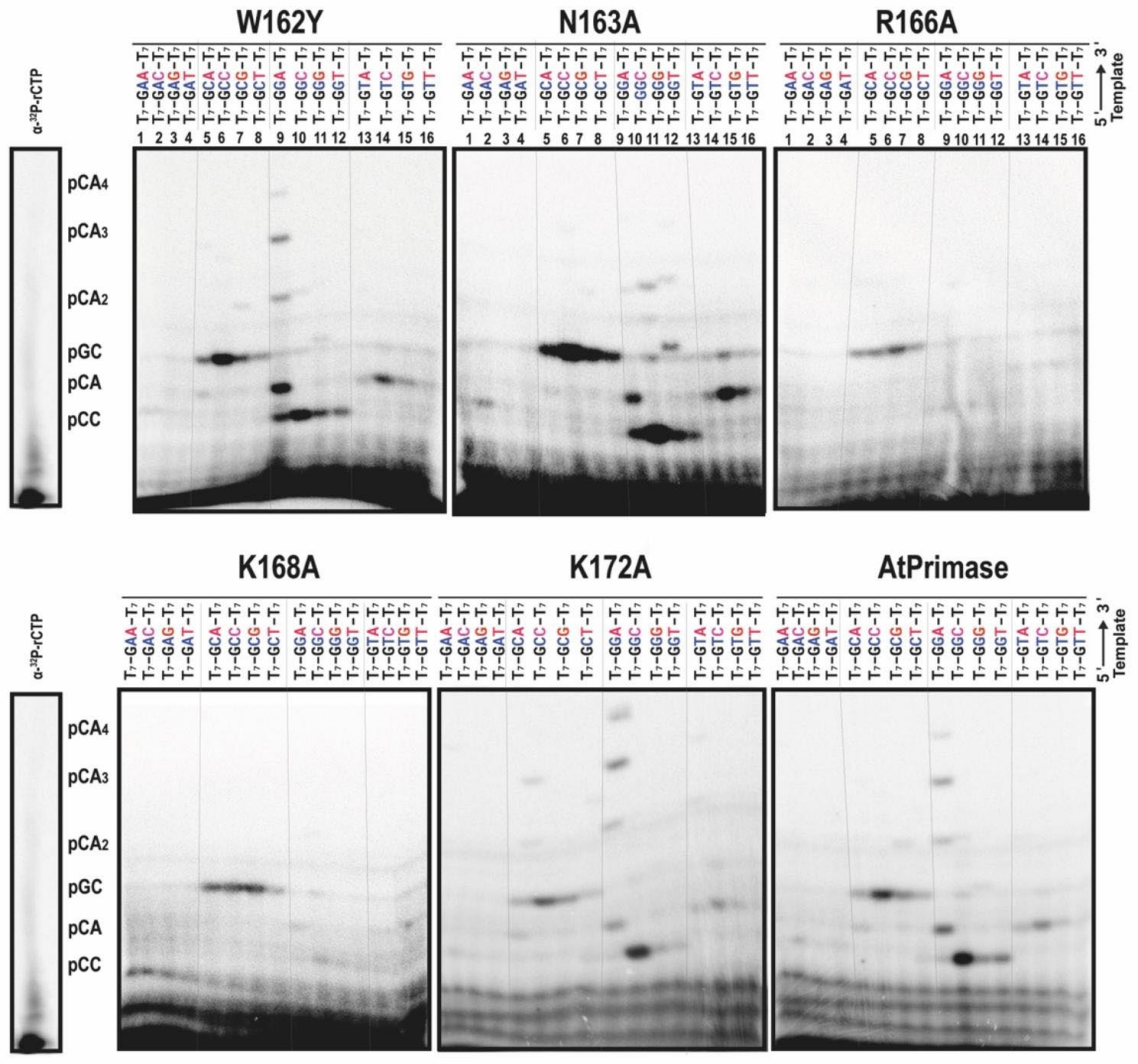
Evaluation of the cryptic pair with point mutants of AtPrimase. Oligoribonucleotide synthesis reactions on substrates in which the cryptic pair was systematically modified to encompass all 16 possibilities. Reaction contained individual oligonucleotides harboring the 5’-T_6_GGNNT_7_--3’ sequence. [α-^32^P]-rCTP is used as radioactive label, because of the marked preference of AtPrimase for CTP as 5´ ribonucleotide

To determine the full extent of the interactions between the point mutants and the cryptic elements of the recognition site in AtPrimase, we tested the entire landscape of variations at the cryptic site. In this experiment, the cryptic 5´-GA-3´ site was systematically changed for every nucleotide, while the G that templates for 5´rCTP was kept constant. AtPrimase mutants W162Y and K172A exhibited a similar pattern of RNA products as wild-type AtPrimase on all tested templates, although AtPrimase-W162Y exhibited a slight increase in formation of the pCC diribonucleotide on a canonical recognition sequence (Fig. 3).

In contrast, mutants R166A and K168A were unable to incorporate ribonucleotides on most of these templates. These mutants synthesize primers only in templates harboring the 5′-GCN-3′ sequence, in which N represents any of the 4 bases. In this sequence these mutants synthetize a pGX diribonucleotide. However, these mutants were unable to extend the initial synthesized diribonucleotide **(**Fig. 3**).** Wild-type AtPrimase and the other point mutants synthesize a pGN diribonucleotide on the same templates, however they were able to extend them. Moreover, mutant N163A showed an increased diribonucleotide synthesis while ribonucleotide synthesis on the canonical template was severely hindered. The latter suggests that residues N163, R166, and K168 are involved in recognizing the cryptic element of the template recognition sequence 5′-(G/C)CGA-3′.

### A chimeric Primase harboring the ZFD of AtPrimase and the RNAPD of T7 Primase is active on the T7 primase recognition sequence

**Fig. 4:**
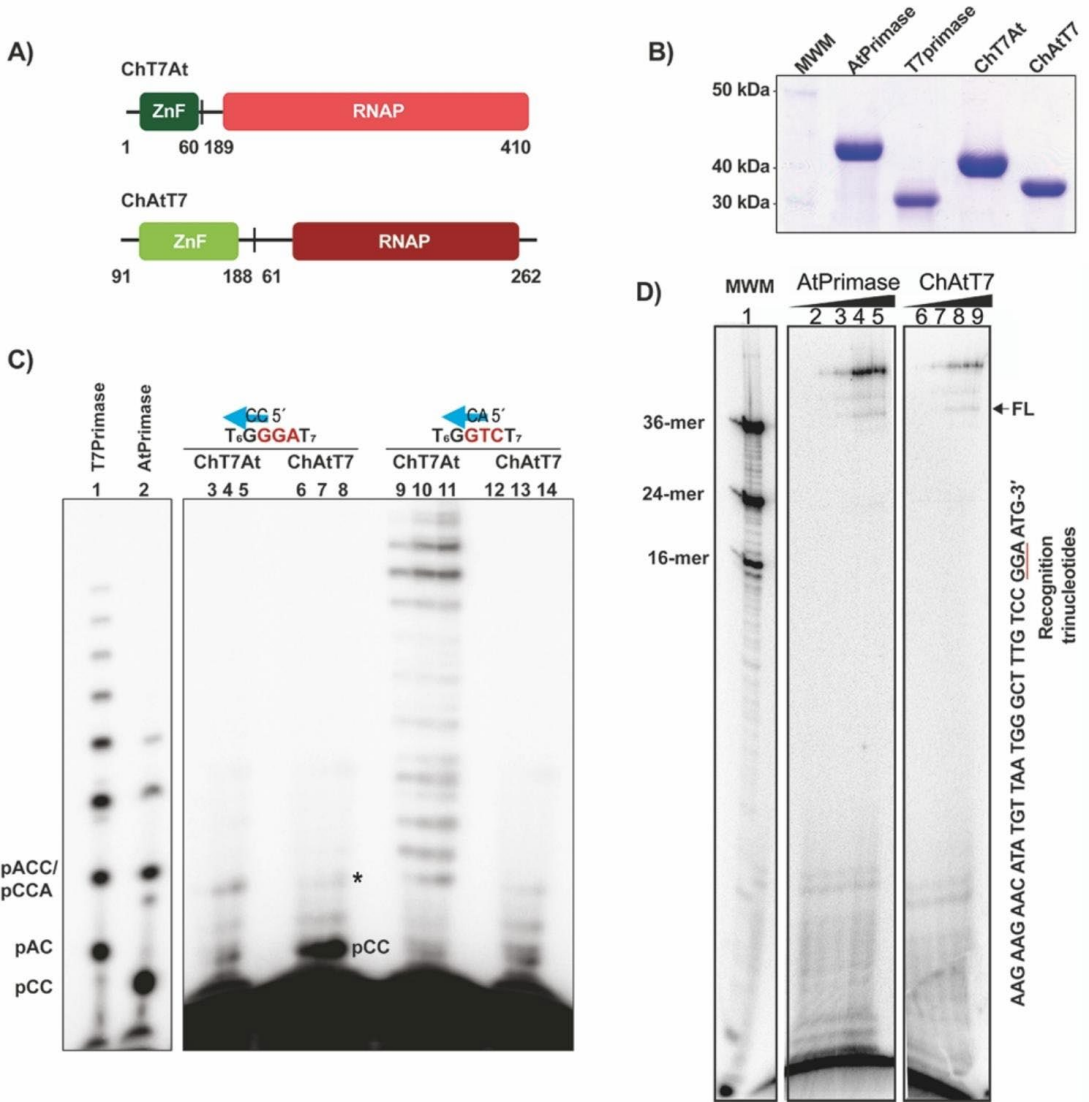
Oligoribonucleotide synthesis by chimeric primases of *A. thaliana* mitochondria and bacteriophage T7. **A)** Representative diagram of the regions used to construct the chimeras between the ZFDs of T7 Primase and AtPrimase. The green blocks represent the ZFDs and the red blocks the RNAP domains. The numbers indicate the amino acids of the wild-type enzymes that were used to construct the chimeras by swapping the ZFD and RNAP domains. **B)** SDS-PAGE gel showing purified chimeric primases. **C)** Denaturing sequencing gel showing oligoribonucleotide synthesis by wild-type AtPrimase and T7 Primase on their canonical templates (lane 1 and 2, respectively), chT7At chimera (lanes 3 to 5 and 9 to 11) and chAtT7 chimera (lanes 6 to 8 and 12 to 14). The identity of the canonical substrates for AtPrimase and T7 Primase is 5’-T6GGGAT7-3’ substrate and 5’-T6GGTCT7-3’ substrate, respectively. The cryptic elements are underlined. The chT7At chimera efficiently synthesizes primers and harbors an increased substrate-independent oligoribonucleotide synthesis activity. This chimera was only able to synthesize primers on the substrate containing the bacteriophage T7-primase recognition trinucleotide. chAtT7 was extremely inefficient, synthesizing a product of maximum size of 4 nucleotides. Primase chimeras were tested at 100, 200 and 400 nM. The ssDNA substrate was used at 5 µM. **D)** Wild-type and chimeric AtPrimases prime organellar DNA polymerase (AtPolIB). Coupled AtPrimase-DNA polymerase reactions on a canonical AtPrimase ssDNA recognition sequence labeled with [α-^32^P]-dATP. AtPolIB, generates DNA:RNA products that correspond to products longer than 36 nts in the presence of increasing concentrations of AtPrimase and ChAtT7 chimera (lanes 2 to 5 and 6 to 9, respectively). The AtPrimase recognition sequence is underlined. In this experiment AtPrimase was present at concentration of 150, 300, and 600 nM whereas AtPolIB was maintained at a fixed concentration of 150 nM

To test the hypothesis that the ZFD of AtPrimase drives template recognition, we constructed two chimeric Primases, one harboring the ZFD of the T7 Primase and the RNAP domain of AtPrimase (ChT7At) and another construct harboring the ZFD of AtPrimase and the RNAP domain of T7 Primase (ChAtT7) (Fig. 4A).

Both chimeric proteins were purified with similar yields and purity than AtPrimase or T7 Primase **(**Fig. 4B**)**. The ChT7At chimera synthesized pAC and pCCA RNA products on the AtPrimase recognition sequence but failed to extend these products to longer RNA primers **(**Fig. 4C, **lanes 3 to 5)**. In contrast, the ChT7At chimera was fully active on a T7 recognition sequence synthesizing ribonucleotides up to 11 nts **(**Fig. 4C, **lanes 9 to 11)**. The ChAtT7 chimera was largely inactive on the T7 recognition sequence and only few pCC products were synthesized (Fig. 4C, **lanes 12 to 14**). In contrast, the ChAtT7 chimera synthesized an abundant amount of the pCC dimer on the AtPrimase recognition sequence and few of those products were extended to an pCCA RNA product and as in the case of the ChT7At these products failed to be extended to tetra-, penta- or longer ribonucleotides **(**Fig. 4C, lanes 6 to 8). The pCC product on the AtPrimase recognition sequence suggest that the ChAtT7 chimera specifically recognizes the AtPrimase sequence.

### ChT7At and ChAtT7 chimeras prime plant Organellar DNA polymerases

We have shown that AtPrimase selectively generate primers that are used by AtPolIs, but those primers are not used by the Klenow Fragment of *E. coli* and T7 DNAP [13]. We were curious to test if the tri ribonucleotide primer (pCCA) synthesized by ChAtT7 **(**Fig. 4C, **lanes 6 to 8 and labeled with an asterisk)** could be used as a primer by AtPolIB **(**Fig. 4D**)**. In this experiment, a single-stranded DNA template containing the 5′-CCGGA-3′ sequence was incubated with increased concentrations of AtPrimase and ChAtT7 in the presence of radioactively labeled dNTPs. A reaction incubated with wild-type AtPrimase and AtPolIB makes for an initial amplification product of 36 nts that is extended to longer products **(**Fig. 4D, **lanes 2 to 5)**, possibly by the terminal transferase activity of AtPolIB [28]. Reactions incubated in the presence of ChAtT7 also resulted in an amplification product of 36nts that are extended to longer primer extension products **(**Fig. 4D, **lanes 6 to 9)**, although the efficiency of the amplification reaction was significantly lower than the one incubated with the wild-type AtPrimase **(**Fig. 4D**)**.

### Individually ZFD and RPD domains of AtPrimase functionally assemble

**Fig. 5:**
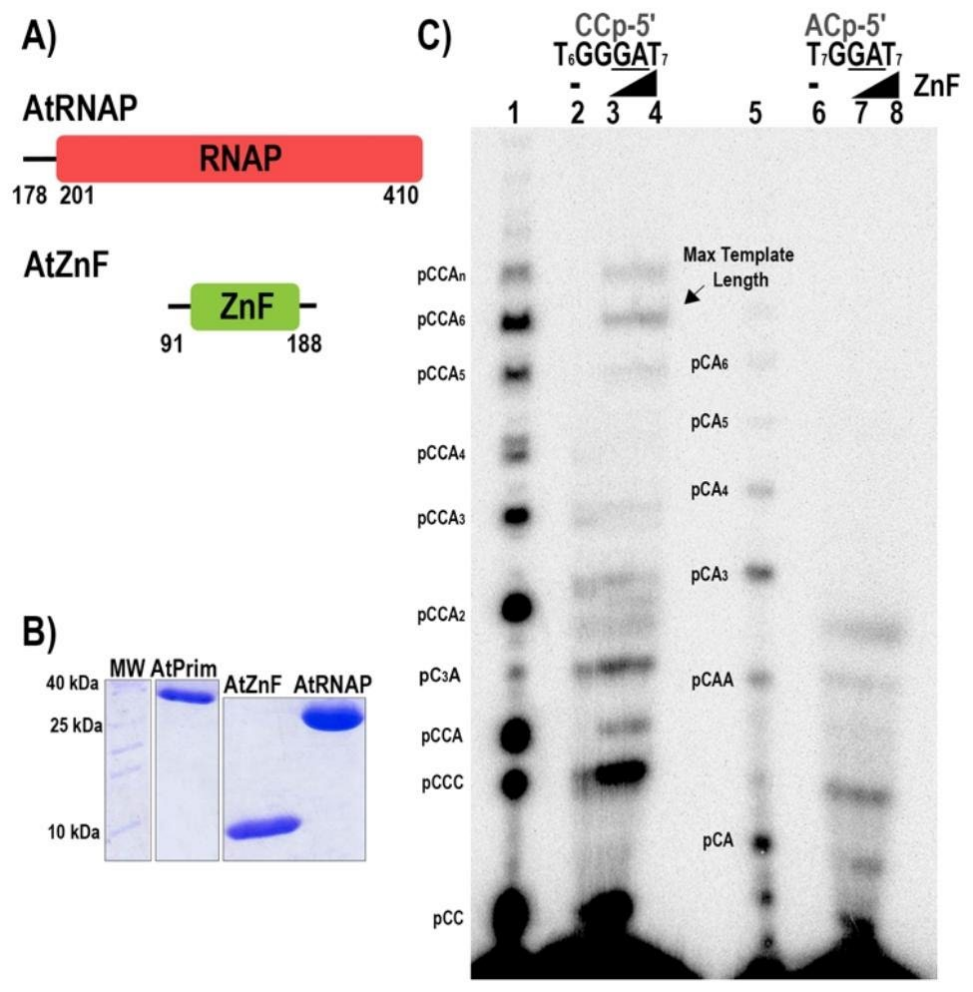
Complementation of AtPrimase subunits results in oligoribonucleotide synthesis **A)** Diagram of the deletion mutants of the primase domain. The numbers below the blocks indicate the position of the residues included in each construct **B)** SDS-PAGE gel showing the purified enzymes used in the primase activity assay. **C**) Complementation assay of activity of deletion mutants of the primase domain. Reaction using AtPrimase on both substrates serve as molecular weight marker. In a substrate with the canonical sequence 5’-T_7_-GGGA-T_7_-3’, the individual AtPrim domains were able to restore their primase activity (lanes 2 to 4). The primase activity of the domains acting individually is severely affected in the substrate with a modified sequence 5´-T_7_-GGA-T_7_-3´ (lanes 6 to 8). AtPrim and AtRPD were used at 2000 nM, AtZnF was used at 2000 and 4000 nM

We individually expressed the ZFD and the RPD of AtPrimase and evaluated if these individually expressed domains can functionally complement each other (Fig. 5).

In this experiment, we use a canonical and a modified AtPrimase recognition sequence in the absence or presence of increasing concentrations of ZFD. The RPD domain by itself lacks the ability to initiate oligoribonucleotide synthesis. However, the addition of increasing concentrations of ZFD result in an increase of synthesized oligoribonucleotides **(**Fig. 5C, **lanes 1 to 8).** The stimulation in oligoribonucleotide synthesis in the presence of ZFD is stronger in reactions containing the wild-type recognition sequence (Fig. 5C, **lanes 1 to 4**) in comparison to reactions assayed on a modified recognition sequence **(**Fig. 5C, **lanes 5 to 8**).

## Discussion

To date, the only characterized primase activity in bifunctional primase-helicases from eukaryotes are those from *Dictyostelium discoideum* (DdTwinkle) and *A. thaliana* (AtTwinkle) [19, 31]. In contrast to the animal primase-helicases, DdTwinkle and AtTwinkle efficiently execute oligoribonucleotide synthesis. AtTwinkle preferentially uses the 5′-(G/C)CGA-3′ sequence to start ribonucleotide synthesis. In contrast to all primases characterized to date, AtTwinkle uses two nucleotides, 5′-GA-3′, as cryptic elements [19]. Given the role of the ZFD of orthologous DNA primases in template recognition, we hypothesized that the ZFD of AtPrimase determines the ability of this enzyme to use two nucleotides as cryptic elements.

We hypothesized that a set of divergent amino acids between T7 and Arabidopsis primases are involved in template recognition **(**Fig. 2**).** Residues W162, N163, R166, K168, and K172 are in an optimal position to interact with ssDNA (Fig. 2). The primase activity of the K172A mutant resulted in the synthesis of RNA products that are similar to those synthesized by wild-type AtPrimase (Fig. 2). Mutants W162Y and N163A increase pCCC ribonucleotide synthesis, which is the expected RNA product of a primase that only recognizes the last base of the cryptic pair, while R166A and K168A mutants strongly decrease ribonucleotide incorporation. These results suggest that residues W162 and N163 are involved in the recognition of two nucleotides as the cryptic element, while residues R166 and K168 abolished the ability of the ZFD to bind to ssDNA. To further determine the full extent of the interactions between the mutants and the cryptic element of the recognition site, we tested variations of the cryptic site. In this experiment, the 5′-GA-3′ cryptic site was consistently changed for each of its sixteen possible sequences. The initial G that templates for C was kept constant using substrates with the 5´-T_7_-GNN-T_7_-3´ sequence (Fig. 3). AtPrimase-K171A exhibited almost identical ribonucleotide synthesis than wild-type AtPrimase on all tested substrates. Interestingly, the R166A and K168A mutants were unable to incorporate ribonucleotides in most of the assayed templates. Only in templates with the 5′-GCN-3′ sequence these mutants synthesize a pGN dinucleotide but were unable to extend it. The N163A mutant exhibited a strong decrease in RNA synthesis on the canonical template sequence, suggesting that this residue participates in the recognition of the cryptic base pair (Fig. 3). Finally, the W162Y mutant exhibited a similar ribonucleotide synthesis with respect to the wild type, but also show an increase in the formation of a product corresponding to the recognition of only one cryptic base. This suggests that this residue is also involved in the use of two bases as cryptic elements. This experiment suggests that residues W162 and N163, are involved in the recognition of the purine pair (GA) as a cryptic element (Fig. 3). Accordingly to this suggestion, Zn^++^ fingers that recognize sequences rich in purines (such as GAG or GAGA) harbor an asparagine residue for the recognition of N_7_ atom of adenosine and the recognition of guanosines is determined by residues like tryptophan of phenylalanine [29, 30] (Fig. 3).

To investigate whether the RNA polymerase domain, adjacent to the ZFD is involved in template recognition, we constructed chimeric enzymes (Fig. 4). These chimeras harbor the ZFD of bacteriophage T7 primase with the RNAP domain of the *A. thaliana* primase, and a chimera assembled by the ZFD of the primase of *A. thaliana* and the RNA polymerase domain of the bacteriophage T7 primase. We dubbed those chimeras as ChT7At and ChAtT7, respectively (Fig. 4). The ChT7At and ChAtT7 chimeras show contrasting results. In one hand, the ChT7At chimera is fully active on a T7 primase recognition sequence, whereas the ChAtT7 chimera only synthesized a pCC dimer on the AtPrimase recognition sequence. A minimal amount of those products was extended to a pCCA trimer, and this chimera was inefficient in extending the initial diribonucleotide and tri ribonucleotide products to longer elongation products (Fig. 4). It is proposed that the ZFD positions the initial diribonucleotide from the active site of the RPD to its N-terminal domain to allow diribonucleotide to 3mers or 4mers [26]. Thus, the high amount of pCC dimer synthesized by the ChAtT7 chimera and the complete activity of the ChT7At chimera on a T7 primase recognition indicates the role of the ZFD in recognizing a cryptic sequence during diribonucleotide synthesis.

As the ChAtT7 chimera synthesizes triribonucleotide primers we were curious to know if those RNA primers could be used by the plant mitochondrial DNAPs, in this case AtPolIB. We found that the primers synthesized by the ChAtT7 chimera are extended by AtPolIB, even when those primers were synthesized by the catalytic domain of bacteriophage T7 primase. During the evolution of plant organelles, the ancestral bacteriophage T7-like DNA polymerase was lost. Plant organelles harbor a DNA polymerase that is non-related to T7 DNA polymerase [31–33]. In contrast, plant Twinkle and T7 helicase-primase are phylogenetically related and share biochemical similitudes [13, 16]. We think that AtTwinkle is able to efficiently synthesize primers for a DNA polymerase that is distant to T7 DNA polymerase, because the ZFD of AtPrimase as an independent module evolved to interact with the Plant Organellar DNA polymerases and execute primer transfer into their active site.

In T7 primase-helicase, the ZFD acts in collaboration with an adjacent RNAP domain rather than with its RNAP domain to execute primer synthesis. To evaluate if the ZFD of AtPrimase could restore the activity of a RNAP domain of AtPrimase we used independently purified ZFD and RNAP domains to evaluate their ability to synthesize ribonucleotides. We found that the RNAP domain and the ZFD function as an active primase, even when they are physically separated (Fig. 5). The latter suggests that AtTwinkle is able to execute priming in *trans* as is the case for T7 primase [18].

**Fig. 6:**
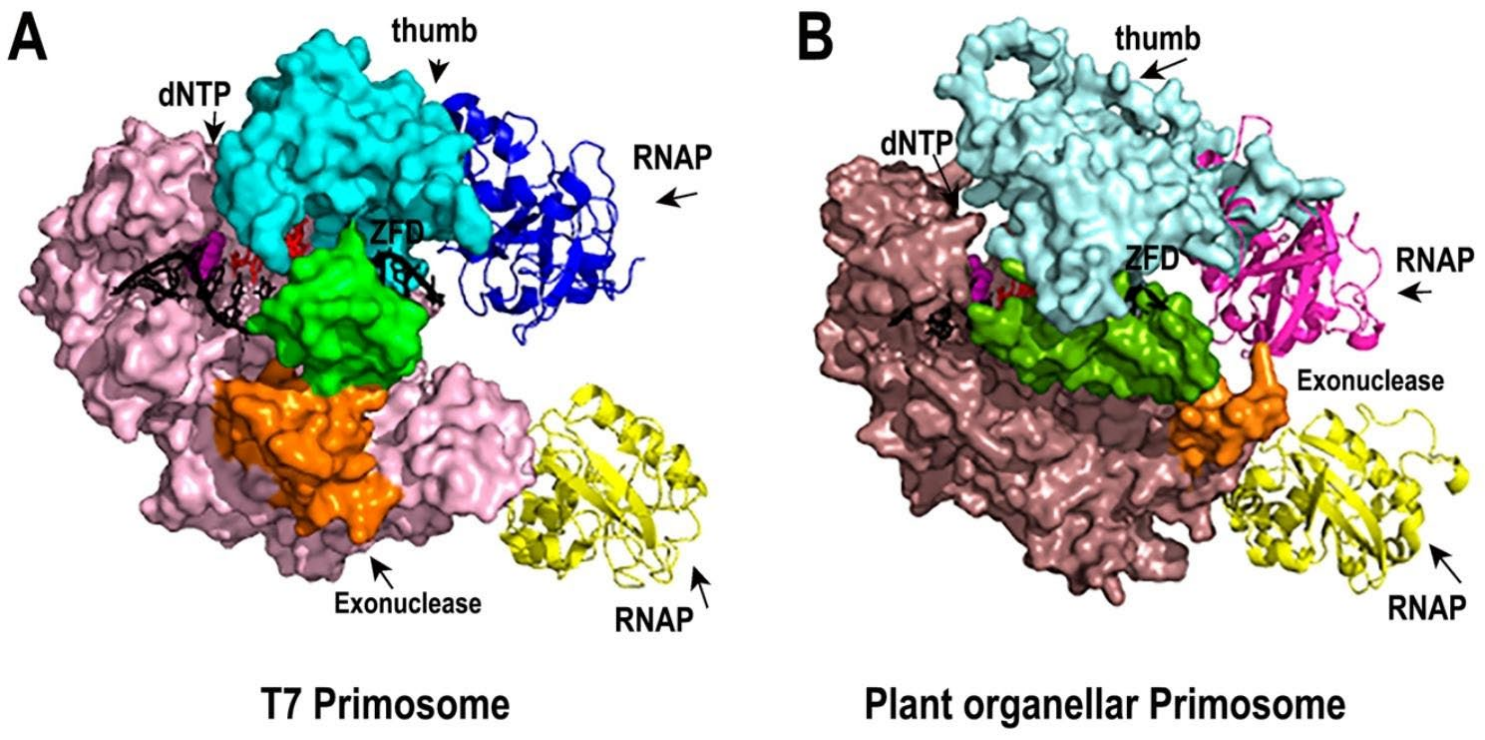
Structural model of the plant organellar primosome. **(A)** Cryo electron structure of the T7 primosome [24]. T7DNAP is draw in a surface representation. The ZFD is green colored and locates at a clef between the thumb subdomain (cyan) and a unique a helix of the exonuclease domain (orange) The RNAP domain of T7 primase (*cis* in blue and *trans* in yellow) interact with the thumb subdomain (blue) and the exonuclease domain, respectively **(B)** Structural model of the proposed plant organellar primosome. The structural model of AtPolIB is in surface representation and colored in light brown. A unique loop of the exonuclease domain is colored in orange and the thumb subdomain is colored in aquamarine. The ZFD of plant mitochondrial AtPrimase is colored in olive and is positioned between a unique insertion (Insertion 1) of the thumb subdomain and an exposed loop of the exonuclease domain (orange). The RNAP domain of AtPrimase (*cis* in magenta and *trans* in yellow) are poised to interact with the thumb subdomain and the exonuclease domain respectively

The electron microscopy structure of T7 DNA Primase and T7 DNAP after priming delivery shows a direct set of interactions between the ZFD of the T7 primase and the exonuclease and thumb subdomain of T7 DNAP, those interactions strongly suggest that the ZFD and T7DNAP have co-evolved to perform specific protein-protein interactions and that an exogenous ZFD could not execute primer delivery (PDB ID: 6n9u) **(**Fig. 6A) [24, 34]. A structural model of AtPolIB with AtPrimase during primer delivery, shows that AtPolIB is predicted to interact with the ZFD of AtPrimase via its insertion 1 and an exonuclease domain a helix, two structural elements that are unique to plant mitochondrial DNAPs [13, 35] **(**Fig. 6B).

## Conclusions

D-loop, theta model, rolling circle, and recombination-dependent replication processes are proposed mechanisms for the replication of plant organellar genomes [36–40]. The recombinogenic nature of plant mitochondrial DNA and the presence of enzymes involved in homologous recombination in plant organelles suggests that plant organellar DNA replication may be accomplished through recombination-dependent replication (RDR) [11, 14]. As an alternative, Morley and coworkers proposed that a mechanism similar to the coordinated leader and lagging-strand replication model of bacteriophage T7 is used to replicate plant mitochondrial DNA [12, 41]. According to this scenario, short ribonucleotides are synthesized by the primase module of AtTwinkle and those ribonucleotides are transferred into the active site of the lagging strand DNA polymerase [13].

Interestingly, plants harbor another primase dubbed PrimPol [42, 43]. In Arabidopsis, AtPrimPol is found in the nucleus, mitochondria, and chloroplast. Human PrimPol reinitiates mitochondrial and nuclear DNA replication at DNA lesions, chain terminating nucleotides or complex structures, like G-quadruplexes, to assure the continuous progression of the replication fork to maintain DNA replication [44–47]. AtPrimPol synthesizes primers that are elongated by organellar DNA polymerases, suggesting that in plant organelles AtPrimPol may participate in the assembling of replication forks as an alternative mechanism to avoid replication fork collapse.

## Methods

### Site directed mutagenesis and chimeric proteins construction

The open reading frame of AtPrimase (residues 92–410) was PCR amplified from a codon optimized AtTwinkle vector using AtPrimase_Nter (5´-aggaaaCATATGaccccggttgatacc) and AtPrimase_Cter (5´-agaagGGATCCttaatagcgtttcgg) oligonucleotides. The PCR product was subcloned between the *Nde* I and *Bam* HI restriction sites (capital letters) of a modified pET19 vector to create the pET19-AtPrimase plasmid. Site-directed mutagenesis was carried out using a modified version of the Q5 Site-Directed Mutagenesis Kit (New England Biolabs). For each point mutants, the pET19-AtPrimase vector was subject to PCR amplification using the oligonucleotides depicted in Table S1. Each PCR product was incubated with T4 kinase and T4 ligase for 30 min and subject to Dpn I treatment for 2 h. 1 µl of the reaction was used to transform chemically competent *E. coli*. The expression plasmid for the primase domain of bacteriophage T7 primase-helicase (residues 1-262) was a gift from Dr. Charles C. Richardson laboratory [21].

AtZnF was constructed using a PCR reaction using the AtPrimase_Nter oligonucleotide in combination with the AtZnF oligonucleotide (5´-ctcttccGGATCCttacggatctgctgaggccaggcc). AtRPD was constructed via PCR amplification using the NtRPD oligonucleotide (5´- aaaggaaaCATATGaccgttgaaggtattgaactg) and the AtPrimase_Cter oligonucleotide. ChT7AT and ChAtT7 chimeric proteins were constructed using the Gene Splicing by Overlap Extension (SOEing) protocol [48]. Detailed explanation of this methodology is presented in supplementary materials. AtZnF, AtRPD, and chimeras were subcloned into a modified pET19 vector between *Nde* I and *Bam* HI restriction sites. The amino acid boundaries for each subdomain of the chimeras are defined in the figure’s legends.

### Protein purification

Protein purification of AtPrimase mutants and chimeric proteins was carried out in an *E. coli* BL21(DE3) strain in LB medium supplemented with 0.1 mM ZnCl_2_ as previously described [13]. Wild-type T7 Primase was expressed and purified as previously described (33).

### Molecular modeling

The homology model of AtPrimase (residues 92 to 410) was built using the molecular operating environment (MOE) platform using the homology model algorithm. The amino acid sequence of AtPrimase was aligned with the amino acid sequence used to solve the crystal structure of bacteriophage T7 primase (PDB ID: 1NUI) [21] using the default settings of the align panel. The MOE platform generated ten homology intermediate models and a final model that is minimized using the CHARMM27 force field.

### Primase reactions

Primase reactions were executed in a buffer containing 40 mM Tris–HCl pH 7.5, 50 mM potassium glutamate, 10 mM MgCl_2_ and 10 mM DTT. Primase reactions contained 100 µM of the indicated NTPs and 10 µCi of [α-^32^P]-NTP (3,000 Ci/mM). Reactions were terminated by adding an equal volume of stop buffer (98% formamide, 0.1% xylene cyanol, 20 mM of EDTA). Reaction products were separated on a 27% denaturing polyacrylamide gel containing 3 M urea. Each primase reaction contained varying amounts of recombinant protein or single-stranded DNA template as indicated in the figure legends. Primase reactions were analyzed by phosphorimaging. Primase reactions were carried out using 5 µM of the synthetic template and 0.5 µM of each recombinant AtPrimase-helicase.

### Polymerase-primase coupled reactions

Coupled primase-DNA polymerase reactions were carried out under the same conditions as the primase reaction, adding 100 µM of dNTPs and labeled with [α-^32^P]-dCTP. AtPrimase and chimeric proteins were present at 150, 300, and 600 nM whereas DNA polymerases were present at 150 nM. The template sequence used in the coupled primase-polymerase assay contained both the AtPrimase and T7 primase recognition sites.

### Electronic supplementary material

Below is the link to the electronic supplementary material.

Supplementary Material 1

Supplementary Material 2

Supplementary Material 3

Supplementary Material 4

## Data Availability

All raw data generated or analyzed during this study are included in this published article.
