# Supplementary material for: The plant organellar primase-helicase directs template recognition and primosome assembly via its zinc finger domain
Source: BMC Plant Biol. 2023 Oct 6;23:467. doi: 10.1186/s12870-023-04477-4 (PMC10557236; doi:10.1186/s12870-023-04477-4)
Supplement: Supplementary file 1 — Supplementary Material 1 [file 12870_2023_4477_MOESM1_ESM.docx]

**Table S1 Oligonucleotides used for site-directed mutagenesis**

**Mutant Upper primer Lower primer**

W162Y AGTGCAACCTacAATTGCTTTCGC AGAACCATCCGGGGCAAT

N163A TGCAACCTGGgcaTGCTTTCGCGG CTAGAACCATCCGGGGCA

R166A GAATTGCTTTgccGGTAAATGTGGTC CAGGTTGCACTAGAACCATC

K168A CTTTCGCGGTgcaTGTGGTCTGA CAATTCCAGGTTGCACTAG

K172A ATGTGGTCTGgcaGGTGGCGTTC TTACCGCGAAAGCAATTC
