# Supplementary material for: The plant organellar primase-helicase directs template recognition and primosome assembly via its zinc finger domain
Source: BMC Plant Biol. 2023 Oct 6;23:467. doi: 10.1186/s12870-023-04477-4 (PMC10557236; doi:10.1186/s12870-023-04477-4)
Supplement: Supplementary file 2 — Supplementary Material 2 [file 12870_2023_4477_MOESM2_ESM.docx]

**Chimeric proteins construction**

To construct the ChT7AT chimera the region corresponding to the ZBD of T7 primase was PCR amplified using oligonucleotides: T7ZNt (5´-aacaacaCATATGgacaattcgcacgattccgat) and T7ZCt (5´-gggtttccgttttgaagccctctctttag). The RPD of ATPrimase was PCR amplified using oligonucleotides AtRPD_N (5´-*ctaaagagagggcttcaaaacggaaaccc*attgaaaaagttgaacgcaaaattaccgttg) and AtRPD_C (5´-ctcttccGGATCCttaaatcggatacggttcagcatc). Both PCR products were gel purified and used as substrates for a second PCR reaction using T7ZNt and AtRPD_C oligonucleotides. The complementary sequence of AtRPD_N oligonucleotide to the T7ZCt oligonucleotide is depicted in italics. To construct the ChAtT7 chimera the region corresponding to the ZFB of AtPrimase was PCR amplified using oligonucleotides AtZNt (5´-aaaggaaaCATATGaccccggttgatacc) and AtZC (5´-ttcaatcggatctgctgaggccaggcc). The RPD of T7 Primase was PCR amplified using oligonucleotides T7RPD_N (5´-*ggcctggcctcagcagatccgattgaa*ggtaaaccaatgacttacaacgtgtg) and T7RPD_N (5´-tgatgatGGATCCtcaaggaatccaaggaccagcattcc).
