# Supplementary material for: The plant organellar primase-helicase directs template recognition and primosome assembly via its zinc finger domain
Source: BMC Plant Biol. 2023 Oct 6;23:467. doi: 10.1186/s12870-023-04477-4 (PMC10557236; doi:10.1186/s12870-023-04477-4)
Supplement: Supplementary file 3 — Supplementary Material 3 [file 12870_2023_4477_MOESM3_ESM.docx]

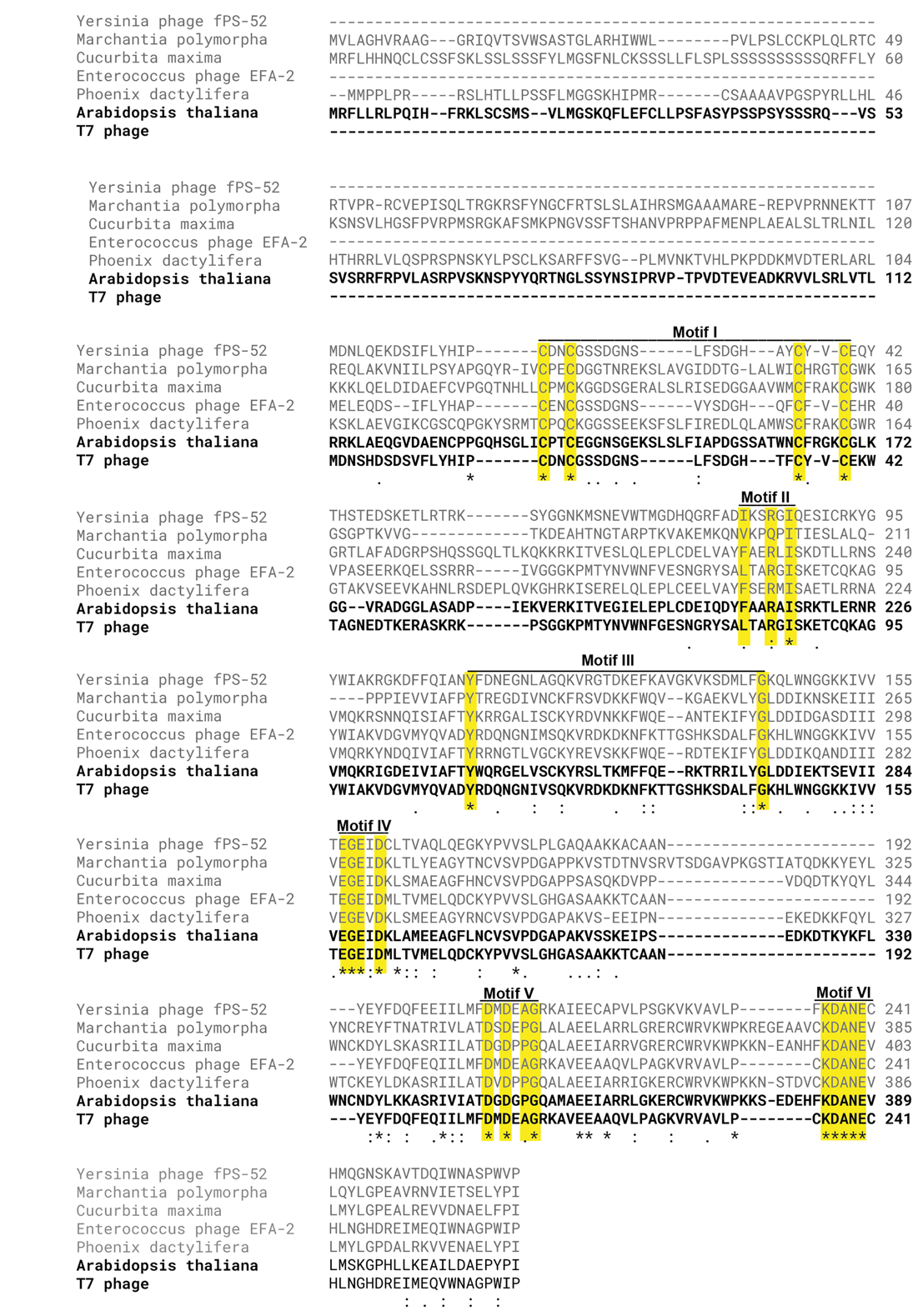


Fig. 1S Multiple amino acid sequence alignment of the primase domains from

representative plants in comparison to primases from T7 and Yersinia phages
