# Supplementary material for: The plant organellar primase-helicase directs template recognition and primosome assembly via its zinc finger domain
Source: BMC Plant Biol. 2023 Oct 6;23:467. doi: 10.1186/s12870-023-04477-4 (PMC10557236; doi:10.1186/s12870-023-04477-4)
Supplement: Supplementary file 4 — Supplementary Material 4 [file 12870_2023_4477_MOESM4_ESM.docx]

Supplementary figures


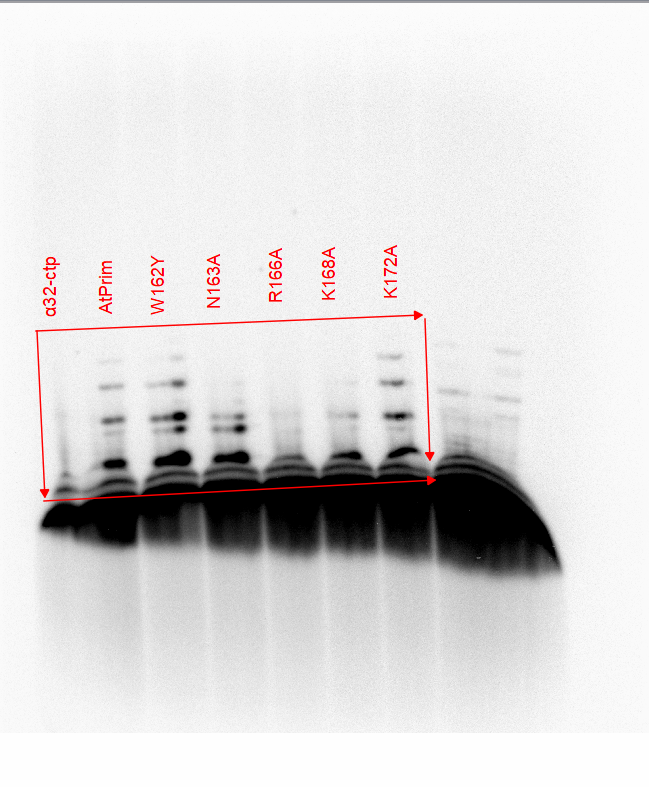


**Original blot used for Fig. 2**


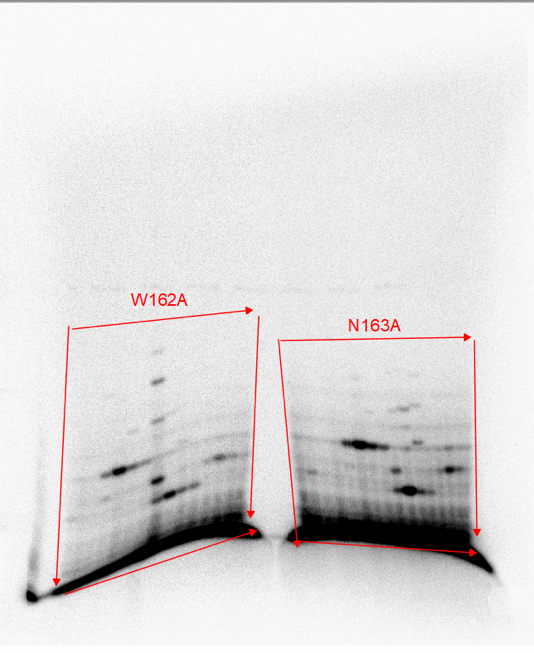

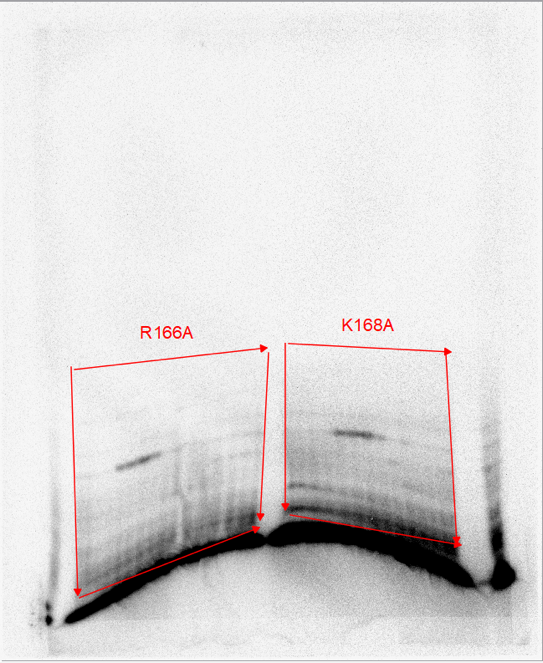


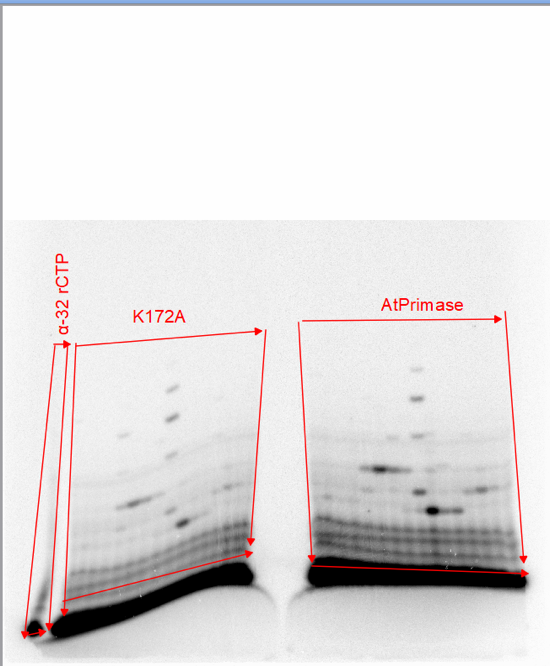


**Original blots used for Fig. 3**


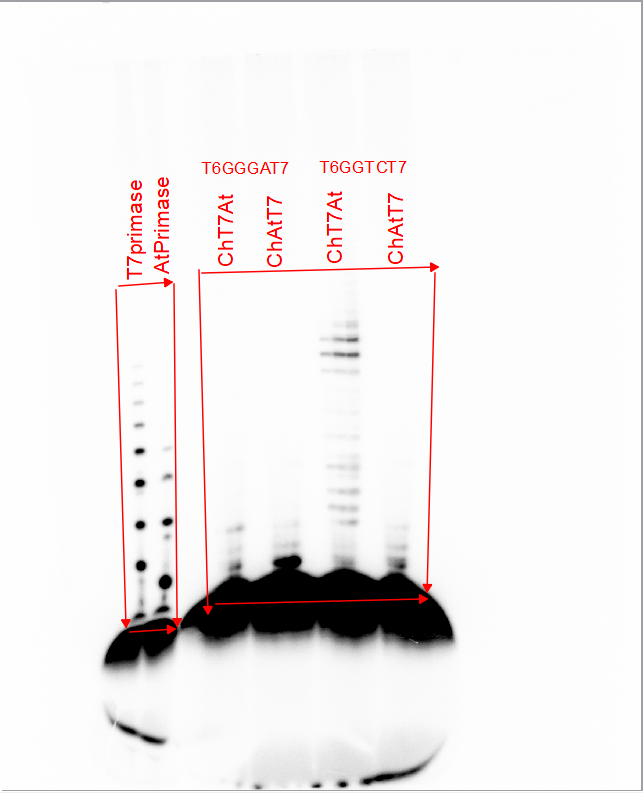

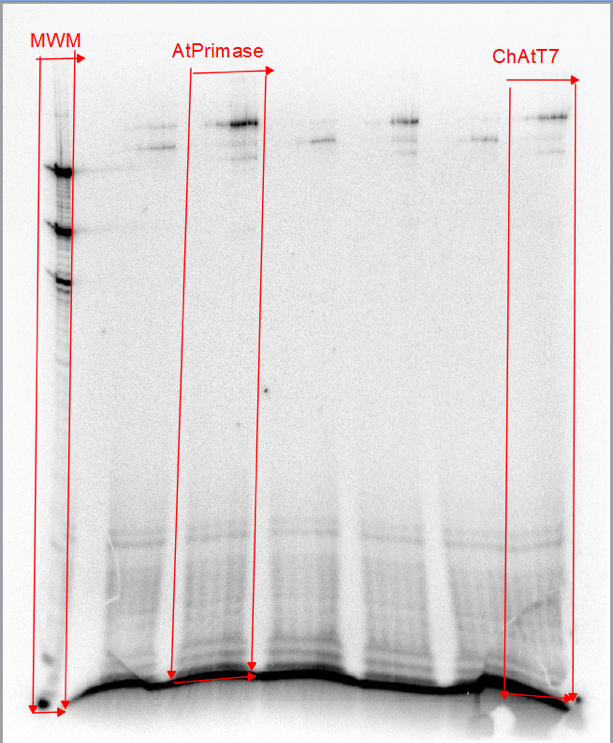


**Original blots used for Fig. 4**


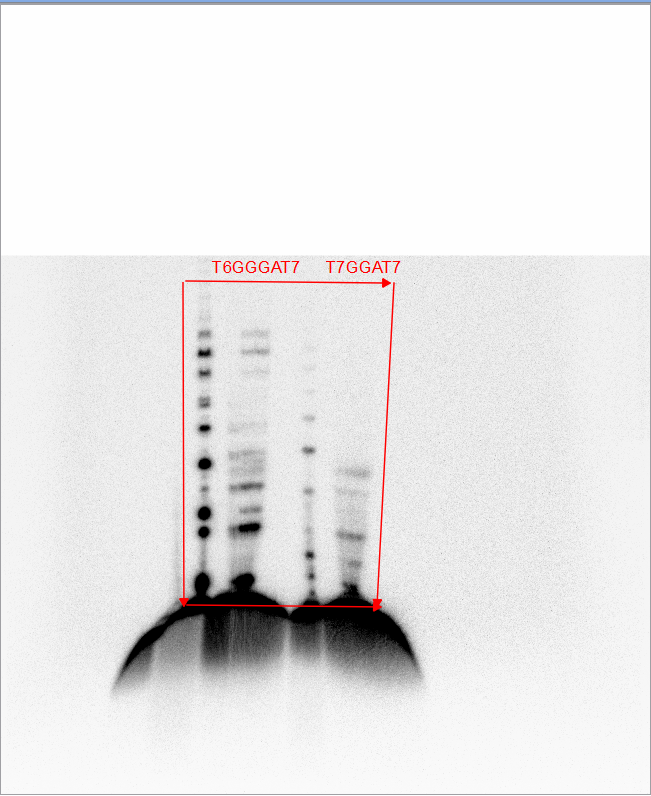


**Original blots used for Fig. 5**
